# Short-chain fatty acids and intestinal macrophage immunometabolism in metabolic syndrome: mechanisms and implications for chronic low-grade inflammation

**DOI:** 10.3389/fimmu.2026.2000672

**Published:** 2026-10-22

**Authors:** Jiaxin Ma, Chen Li, Hongjun Lou

**Affiliations:** Heilongjiang University of Chinese Medicine, Harbin, China

**Keywords:** chronic low-grade inflammation, immunometabolism, intestinal macrophages, metabolic syndrome, short-chain fatty acids

## Abstract

Chronic low-grade inflammation (CLGI) is a major feature of metabolic syndrome (MetS), and the intestinal mucosa is an important site where microbial metabolites interact with host immunity. Intestinal lamina propria macrophages (LpMs) maintain mucosal tolerance, barrier homeostasis, and antimicrobial defense, and their phenotype and metabolism are altered in obesity and related metabolic disturbances. Short-chain fatty acids (SCFAs), generated mainly by microbial fermentation of dietary fiber, influence macrophage function through intracellular metabolism, G protein-coupled receptor signaling, and histone deacetylase inhibition. Butyrate is the best-characterized SCFA in this context and regulates glycolysis, mTOR-associated metabolism, antimicrobial responses, and HDAC-dependent inflammatory programs. Acetate and propionate also affect inflammasome activity, cytokine production, receptor signaling, and cellular metabolism. These mechanisms connect microbial metabolism with intestinal inflammation, epithelial barrier function, and systemic metabolic homeostasis. In obesity and insulin resistance, colonic macrophage recruitment and metabolic remodeling are closely associated with disease progression, whereas macrophage-related effects in metabolic dysfunction-associated steatotic liver disease and hypertension involve broader gut–liver, vascular, and renal pathways. Human studies of dietary fiber, microbiota-directed interventions, and SCFA delivery show variable cardiometabolic effects, with outcomes influenced by formulation, route, and baseline metabolic state. Given that most current mechanisms are derived from extraintestinal macrophages and *in situ* evidence from the human gut remains scarce, this narrative review summarizes the homeostatic biology of intestinal macrophages, the pathways through which SCFAs regulate their immunometabolism, the context-dependent relevance of this axis across MetS components, and the barriers to clinical translation.

## 1 Introduction

Metabolic syndrome (MetS) is increasingly prevalent worldwide (1). Its main components include central obesity, elevated fasting glucose, dyslipidemia, and hypertension (2). These disorders are closely associated with chronic low-grade inflammation (CLGI), which is persistent and less intense than acute inflammatory responses (3). Metabolic dysfunction-associated steatotic liver disease (MASLD) is discussed here as a closely related metabolic condition rather than a diagnostic component of MetS (2, 4).

Studies of chronic low-grade inflammation in MetS have historically focused on adipose tissue macrophages (ATMs), particularly their recruitment and functional polarization during obesity (5). These studies have defined important mechanisms of local adipose inflammation, but they do not fully explain the upstream signals that sustain systemic immune-metabolic dysfunction. With the development of the metabolic endotoxemia concept, attention has increasingly shifted from peripheral metabolic tissues to the intestine, where dysbiosis, impaired barrier function, and microbial metabolites can shape both mucosal and systemic inflammation (6, 7). In a gestational diabetes model, reduced SCFA availability was accompanied by proinflammatory ATM polarization, illustrating how changes in gut-derived metabolites can influence macrophage responses beyond the intestine (8).

Against this background, SCFAs are of particular interest because they connect microbial fermentation of dietary fiber with host immune and metabolic regulation (9). Previous reviews have addressed SCFAs in metabolic disease and inflammation and have summarized the biology of intestinal mononuclear phagocytes (10, 11). Here, we focus on intestinal macrophages and integrate their homeostatic biology with SCFA metabolism, receptor signaling, epigenetic regulation, barrier function, and metabolic disease. Mechanistic studies in extraintestinal macrophages and other immune cells are included where they clarify pathways that may operate within the intestinal mucosa, while tissue context and local SCFA exposure are considered throughout the review.

For this narrative review, PubMed, Web of Science, and Scopus were searched from database inception through August 2026 using combinations of terms related to SCFAs, intestinal macrophages, macrophage immunometabolism, MetS, and SCFA receptors and signaling pathways. English-language primary studies were prioritized for mechanistic interpretation, with reviews used for background and to identify additional reports. Literature was selected according to relevance to SCFA biology, intestinal macrophages, and metabolic disease. Evidence assessment considered the species, cell or tissue model, measured outcomes, and relevance to intestinal macrophages. Direct evidence in intestinal macrophages was distinguished from findings in other models. Gene and protein expression data were considered separately from functional assays and metabolic measurements. These distinctions guided the narrative interpretation of evidence directness and relevance. No formal evidence-grading framework, PRISMA-based selection process, or meta-analysis was used.

## 2 Intestinal macrophages in homeostasis and the local environment

### 2.1 Monocyte differentiation and homeostatic functions

In adult mice, circulating Ly6C^hi^ monocytes continuously enter the colonic lamina propria and mature into tissue macrophages (12, 13). Transcriptomic analysis of this maturation continuum identified 1,004 genes that changed by more than twofold between newly arrived Ly6C^hi^ monocytes and mature CX3CR1^hi^ colonic macrophages (14). Genes associated with recruitment and inflammatory signaling, including *Ccr2*, *Trem1*, and *Myd88*, decreased during maturation, whereas homeostatic genes such as *Il10*, *Mrc1*, *Mertk*, *Cd163*, and *Itgax* increased (14). Human intestinal macrophages show weak inflammatory responses to microbial ligands while retaining efficient phagocytic and bactericidal activity (15). Human phenotypic and single-cell studies likewise identify CCR2-high inflammatory or intermediate populations and more mature CD163/CD206-associated populations (16–20). Together, these data illustrate progressive tissue adaptation of intestinal macrophages, with TGF-β receptor signaling contributing to the colonic differentiation program in mice (14). **Figure 1** illustrates colonic macrophage maturation and the local microenvironment.

**Figure 1:**
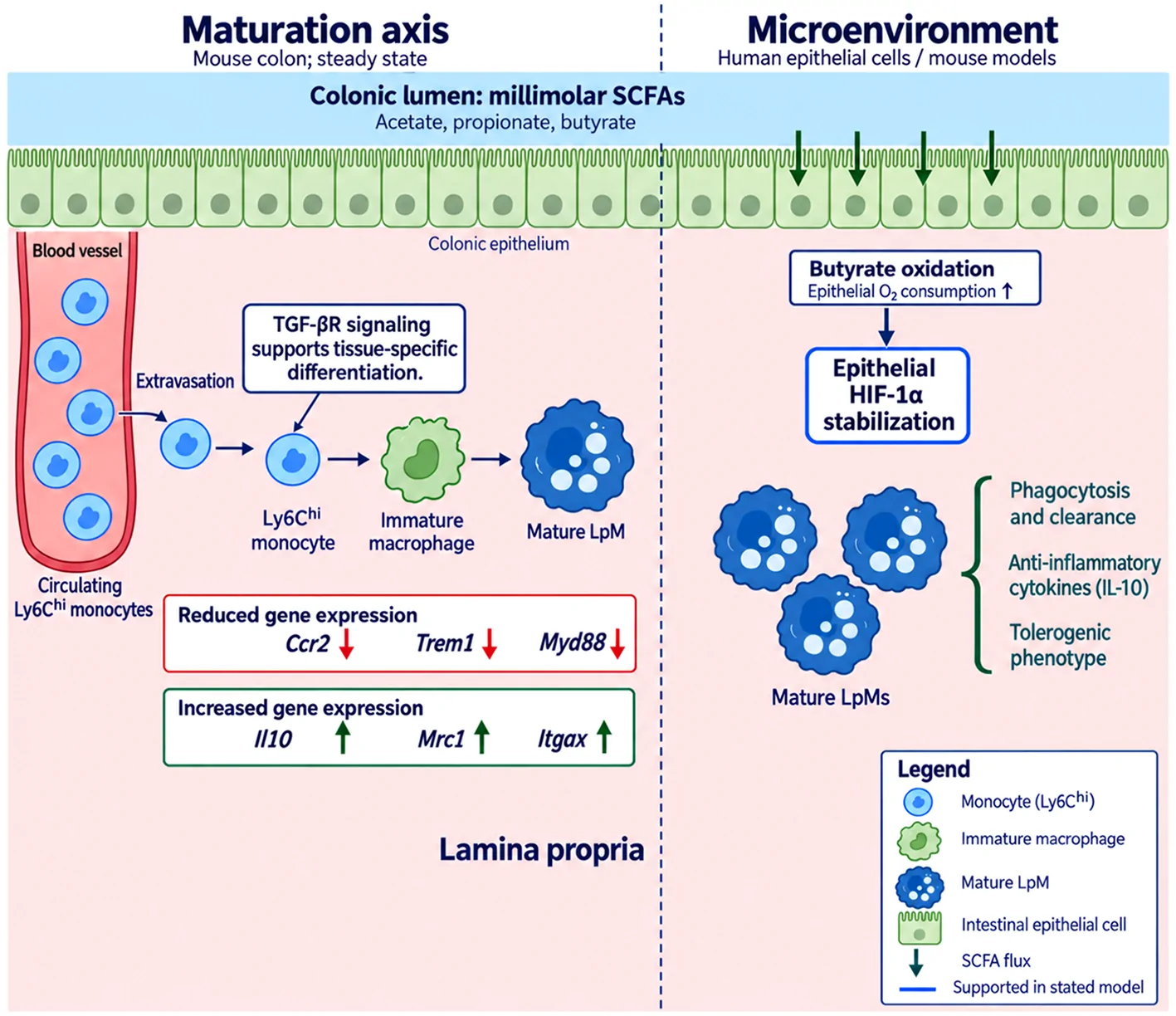
Maturation and local environment of intestinal lamina propria macrophages. The maturation axis summarizes monocyte-to-macrophage differentiation in the colon, while the microenvironment panel illustrates luminal SCFAs, epithelial metabolism, the mucosal oxygen gradient, and the homeostatic functions of mature LpMs.

This restrained inflammatory program limits unnecessary responses to commensal microorganisms while preserving host defense (15). During obesity and other inflammatory states, recruited monocyte-derived macrophages accumulate and homeostatic programs are remodeled (21–24). Mouse obesity studies provide the clearest link between these intestinal macrophage changes and systemic metabolic inflammation, whereas human studies currently define cellular phenotypes more extensively than macrophage metabolism (21–24). Human-relevant models, including iPSC-derived macrophages and intestinal co-culture systems, may help bridge this gap (25, 26).

### 2.2 Anatomical niches and local SCFA exposure

Luminal, mucosal/interstitial, portal, and peripheral SCFA measurements represent distinct exposure compartments (27–29). In the classic human study by Cummings et al. (27), total SCFA concentrations in colonic contents were approximately 80–140 mmol/kg, declining from 131 ± 9 mmol/kg in the cecum to 80 ± 11 mmol/kg in the descending colon. Acetate was the predominant SCFA, whereas propionate and butyrate were present at lower, still millimolar concentrations; the overall SCFA pool and the relative contribution of butyrate decreased distally along the colon (27). The changing molar ratios across colonic contents, portal blood, hepatic blood, and peripheral blood were consistent with substantial epithelial uptake of butyrate and hepatic extraction of propionate (27). Total SCFA concentrations fell to 375 ± 70 μmol/L in portal blood, 148 ± 42 μmol/L in hepatic blood, and 79 ± 22 μmol/L in peripheral blood (27). More recent measurements in the fasted proximal colon confirm that luminal SCFAs remain in the millimolar range (30). Avery et al. (28) further showed enrichment of SCFAs in colonic interstitial fluid relative to plasma, indicating that the mucosal compartment is metabolically distinct from the circulation. SCFAs also influence the local oxygen environment: epithelial butyrate oxidation increases epithelial oxygen consumption and stabilizes HIF, thereby supporting barrier function (31).

LpMs reside beneath the epithelium and may encounter SCFAs after these metabolites cross the mucus and epithelial layers (32, 33). The exact pericellular concentration around LpMs remains unknown, but the intestinal mucosa is characterized by a steep radial oxygen gradient and high local metabolic activity (28, 29, 34). Mature LpMs combine restrained inflammatory responsiveness with efficient phagocytic and antimicrobial activity, a state that is not adequately captured by a simple M2 designation (15, 18). Muscularis macrophages occupy a deeper anatomical niche, interact with enteric neurons through BMP2-CSF1 signaling, regulate gastrointestinal motility, and participate in gut-brain neuroimmune pathways (35, 36). **Table 1** compares the biological characteristics and local environments of intestinal and extraintestinal macrophage populations.

**Table 1:** Biological characteristics and local environments of major macrophage populations.

Characteristic	Intestinal LpMs	Muscularis macrophages	Adipose ATMs	Kupffer cells
Developmental origin	Adult mouse colon: mainly replenished by CCR2-dependent Ly6C^hi^ monocytes. Human subset relationships are defined from phenotype and single-cell trajectories rather than lineage tracing (12, 13, 18).	Long-lived embryonic populations with adult monocyte contribution that increases during tissue disturbance (35).	Mixed resident and monocyte-derived populations. Recruited macrophages increase in obesity (5, 37, 38).	Embryonically seeded resident Kupffer cells. Monocyte-derived macrophages accumulate and can repopulate the niche during liver injury (39).
Surface markers	Mouse mature LpMs express CX3CR1^hi^, MHCII+, and CD64 +. Human subsets express HLA-DR and CD64, while CD163 and CD206 vary by subset and state (12, 16–18).	Mouse populations express CX3CR1 and MHCII and include neuron-associated, BMP2-producing programs. Phenotype varies by niche and state (35).	Obesity-associated and lipid-associated subsets can express TREM2, CD9, GPNMB, and CD36. These are not universal ATM markers (37, 38).	Resident Kupffer cell programs include CLEC4F, TIMD4, and VSIG4 in mice. TIMD4 and VSIG4 also identify resident-like human subsets, with changes during disease (39).
SCFA exposure	Luminal SCFAs are present in the high millimolar range, approximately 80–140 mmol/kg across the human colon, with a proximal-to-distal decline (27, 30). Colonic interstitial SCFAs are enriched relative to plasma (28); the concentration immediately surrounding LpMs is unknown.	The muscularis is a deeper intestinal compartment; local interstitial SCFA concentration has not been measured directly.	Exposure is mainly systemic or peripheral; adipose interstitial SCFA concentrations are poorly characterized.	Portal exposure to gut-derived SCFAs exceeds peripheral exposure (27); local concentrations around Kupffer cells remain poorly characterized.
Oxygen environment	The intestinal mucosa displays a steep radial oxygen gradient (34); oxygen tension varies across the tissue niche.	Oxygen conditions vary across the muscularis niche and have not been defined at single-cell resolution in the cited study (35).	Adipose tissue becomes locally hypoxic during obesity, although cell-specific oxygen tension varies by depot and disease state.	Kupffer cells occupy the sinusoidal liver niche, where oxygen availability follows the hepatic lobular gradient.
Main functions	Maintain intestinal immune tolerance, clear pathogens, and support barrier integrity (13, 15).	Communicate with enteric neurons and regulate gastrointestinal motility (35).	Regulate adipose tissue lipid metabolism and homeostasis (37).	Clear foreign particles and contribute to hepatic immune tolerance (39).
Disease-related changes	Obesity is associated with expansion of inflammatory colonic macrophages, changes in resident subsets, barrier dysfunction, and metabolic endotoxemia (21–24).	Muscularis macrophages participate in gut-brain neuroimmune processes (36); their role in MetS is less well characterized.	Obesity is associated with expansion of recruited inflammatory and lipid-associated macrophage states and increased adipose inflammation (5, 37, 38).	MASLD is associated with changes in resident Kupffer cells and recruitment of monocyte-derived macrophages. Portal microbial products can contribute to hepatic inflammation (39, 40).

SCFA, short-chain fatty acid; MetS, metabolic syndrome; LpM, lamina propria macrophage; ATMs, adipose tissue macrophages; MASLD, metabolic dysfunction-associated steatotic liver disease.

## 3 Intestinal barrier dysfunction and macrophage changes in metabolic syndrome

### 3.1 Colonic macrophages in obesity

Colonic macrophages undergo marked transcriptional, functional, and metabolic remodeling during obesity (21–24). Transcriptomic profiling, metabolite measurements, ECAR/OCR analysis, and isotope tracing capture different aspects of this remodeling and are complementary rather than interchangeable.

In high-fat diet (HFD)-fed mice, colonic lamina propria macrophages increase in number and include populations with lipid-associated macrophage features, including expression of *Trem2*, *Cd9*, *Gpnmb*, *Cd36*, *Fabp4*, and *Fabp5* (23). The same study reported altered lipid handling, greater phagocytic and efferocytic activity, and higher expression of glycolysis-related genes (23). CCL2/CCR2-dependent recruitment also contributes to inflammatory macrophage accumulation, and *Ccr2* deficiency improved insulin sensitivity in HFD-fed mice (21). Myeloid *Brd4* deletion reduced *Ccr2*/*Ccr5* expression, colonic macrophage infiltration, and metabolic endotoxemia in HFD-fed mice (41). Together, these findings indicate broad transcriptional and functional remodeling of colonic macrophages; higher expression of glycolysis-related genes is consistent with altered glucose metabolism, although direct flux measurements in intestinal macrophages remain sparse. Beyond the lamina propria, HFD feeding also promotes CCR2-dependent monocyte recruitment to the muscularis, with associated enteric neuroimmune remodeling and colonic dysmotility (42).

Human studies similarly show inflammatory remodeling of the intestinal macrophage compartment (24). In people with obesity, sampling along the gastrointestinal tract identified more proinflammatory macrophage subsets together with an increase in circulating intermediate monocytes (24). Metabolic characterization is more detailed in experimental models: in HFD-fed mice, colonic macrophage activation is associated with interferon-response and mitochondrial programs involving mTOR, and local mTOR inhibition reduces inflammatory macrophage abundance while improving metabolic measures (22). TREM2-dependent lipid handling and mTOR/PPARγ signaling in lipid-associated macrophages provide a related framework for understanding how nutrient sensing shapes macrophage states across metabolic tissues (37, 38).

These observations place mTOR at the intersection of inflammatory and homeostatic macrophage metabolism. Fritsch et al. (43) showed that steady-state colonic macrophages support epithelial renewal through an mTORC1-dependent arginase-1 and polyamine pathway, and macrophage *Tsc2* loss increased this metabolic support. Thus, mTOR can support distinct macrophage functions according to the tissue state. How changes in local SCFA availability intersect with these context-dependent mTOR programs in intestinal macrophages remains to be resolved.

### 3.2 Barrier dysfunction, metabolic endotoxemia, and SCFA availability

Obesity is associated with impaired intestinal barrier function (44). Reduced expression of tight-junction proteins such as occludin, claudin-1, and ZO-1, together with changes in the mucus layer and antimicrobial defenses, can increase intestinal permeability and allow luminal lipopolysaccharide (LPS) to enter the portal circulation (44). In diet-induced obese mice, Lam et al. (44) reported a 38% reduction in proximal-colon transepithelial resistance, a 40% decrease in ZO-1 mRNA, and a 6.6-fold increase in TNF-α mRNA. Lower abundance of butyrate-producing bacteria and reduced SCFA availability may further impair epithelial energy metabolism and barrier integrity (10, 45). Once the barrier is compromised, LPS can activate TLR4/MyD88/NF-κB signaling and increase TNF-α, IL-6, MCP-1, and IL-1β (44, 46). These mediators can disrupt IRS-1/PI3K/Akt/GLUT4 signaling and contribute to insulin resistance (44). In obesity and MetS-associated states, modest elevations in circulating LPS have been associated with persistent low-grade inflammation rather than the acute systemic response seen in sepsis (7, 46).

Gut dysbiosis and altered SCFA production are associated with intestinal barrier dysfunction (10, 47). Lower intake of fermentable fiber can reduce microbial SCFA production, while expansion of Gram-negative bacteria may increase the luminal LPS burden (9, 46). Fecal SCFA concentrations reflect the combined effects of microbial production, epithelial absorption, and host utilization (32, 48). Human studies of fecal SCFAs and metabolic disorders report heterogeneous associations (49). Higher fecal SCFAs have been associated with obesity, hypertension, lower microbial diversity, and greater intestinal permeability in humans (47), illustrating why fecal abundance does not necessarily parallel tissue availability (48, 50, 51). Accordingly, luminal, mucosal/interstitial, portal, and peripheral measurements provide complementary information about SCFA exposure and should be interpreted as separate compartments (27–29). **Figure 2** summarizes these relationships.

**Figure 2:**
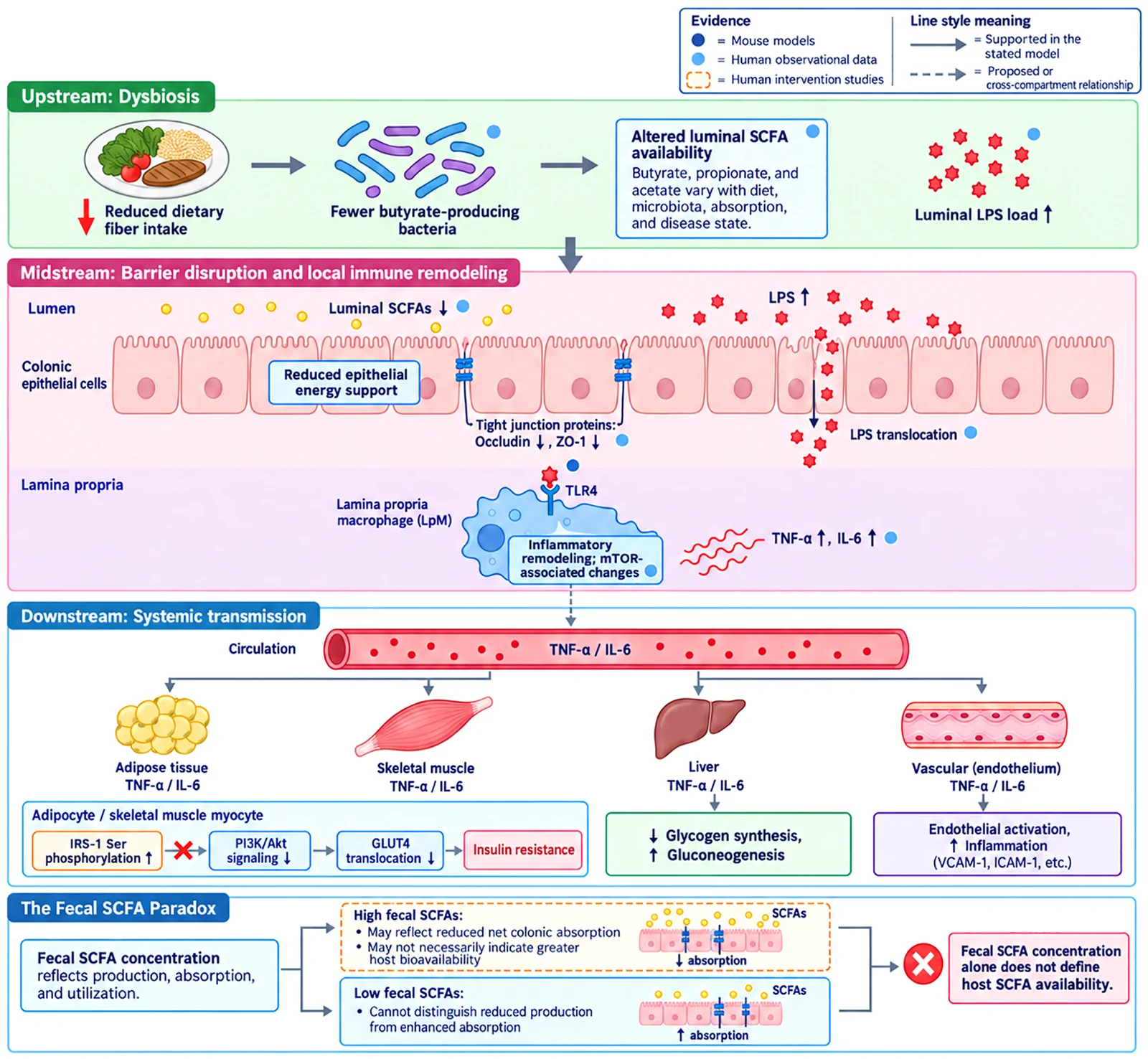
Gut dysbiosis, barrier dysfunction, and systemic metabolic consequences in MetS. Reduced fermentable substrate availability and changes in SCFA-producing bacteria can alter luminal SCFA production, while barrier disruption increases LPS translocation and inflammatory signaling. The lower panel illustrates why fecal SCFA concentrations reflect the balance between production and absorption rather than tissue exposure. Dashed arrows denote proposed cross-compartment relationships.

### 3.3 Gut microbiota dysbiosis and metabolic consequences

Intestinal barrier dysfunction and metabolic endotoxemia link gut microbial changes to peripheral insulin resistance (10, 52). Animal transfer studies provide strong causal evidence that microbiota composition can shape metabolic phenotypes (53, 54), while human interventions show that modifying the microbiota can alter selected metabolic outcomes (55–57).

Ridaura et al. (53) transplanted fecal microbiota from obesity-discordant twins into germ-free mice. Mice that received microbiota from the twin with obesity developed greater adiposity than mice that received microbiota from the lean twin, despite the same diet (53). During cohousing, Bacteroidetes from lean-microbiota recipients colonized mice carrying obese-donor communities and reduced the obese phenotype (53). This effect depended on dietary fiber (53). Tran et al. (54) also found that Western diet-induced adipose inflammation required a complex microbiota. Germ-free or antibiotic-treated mice became obese but showed much less adipose inflammation than conventionally colonized animals (54).

Human intervention studies indicate that changing the intestinal microbiota can modify metabolic outcomes (55–57). In 18 men with MetS, Vrieze et al. (55) found that lean-donor microbiota infusion increased insulin sensitivity at 6 weeks and increased the abundance of butyrate-producing taxa. In a pilot double-blind randomized trial, Piwchan et al. (56) reported improvements in HOMA-IR, fasting glucose, and hs-CRP at 6 weeks after FMT, although effects were not consistent across outcomes at 12 weeks. A meta-analysis also reported modest benefits for selected metabolic measures (57). Together, these studies support a microbiota-dependent component of metabolic regulation, while the metabolites and cellular pathways responsible for individual clinical responses remain incompletely defined.

SCFAs may account for part of the metabolic response to microbiota manipulation, but they act in several cell types (10, 58–63). Studies have examined FFAR2, FFAR3, and HCAR2 signaling, HDAC inhibition, epithelial barrier regulation, GLP-1 release from enteroendocrine cells, T-cell differentiation, and macrophage responses (10, 58–63). The cell type used in each experiment is therefore important when interpreting the mechanism.

Clinical responses to FMT vary with donor engraftment, baseline fermentation capacity, study population, and follow-up duration (57, 64). This heterogeneity has shifted attention toward donor-recipient compatibility and metabolic context as important determinants of treatment response.

## 4 SCFA mechanisms in macrophage immunometabolism

Butyrate has the largest mechanistic evidence base in macrophage immunometabolism (59, 65), while acetate and propionate also influence macrophage metabolism and inflammatory responses (66–71). Mechanistic work spans intestinal and extraintestinal macrophages as well as other immune cells, allowing complementary views of intracellular metabolism, receptor signaling, and epigenetic regulation. **Figure 3** summarizes the major pathways, and **Table 2** lists representative experimental studies.

**Figure 3:**
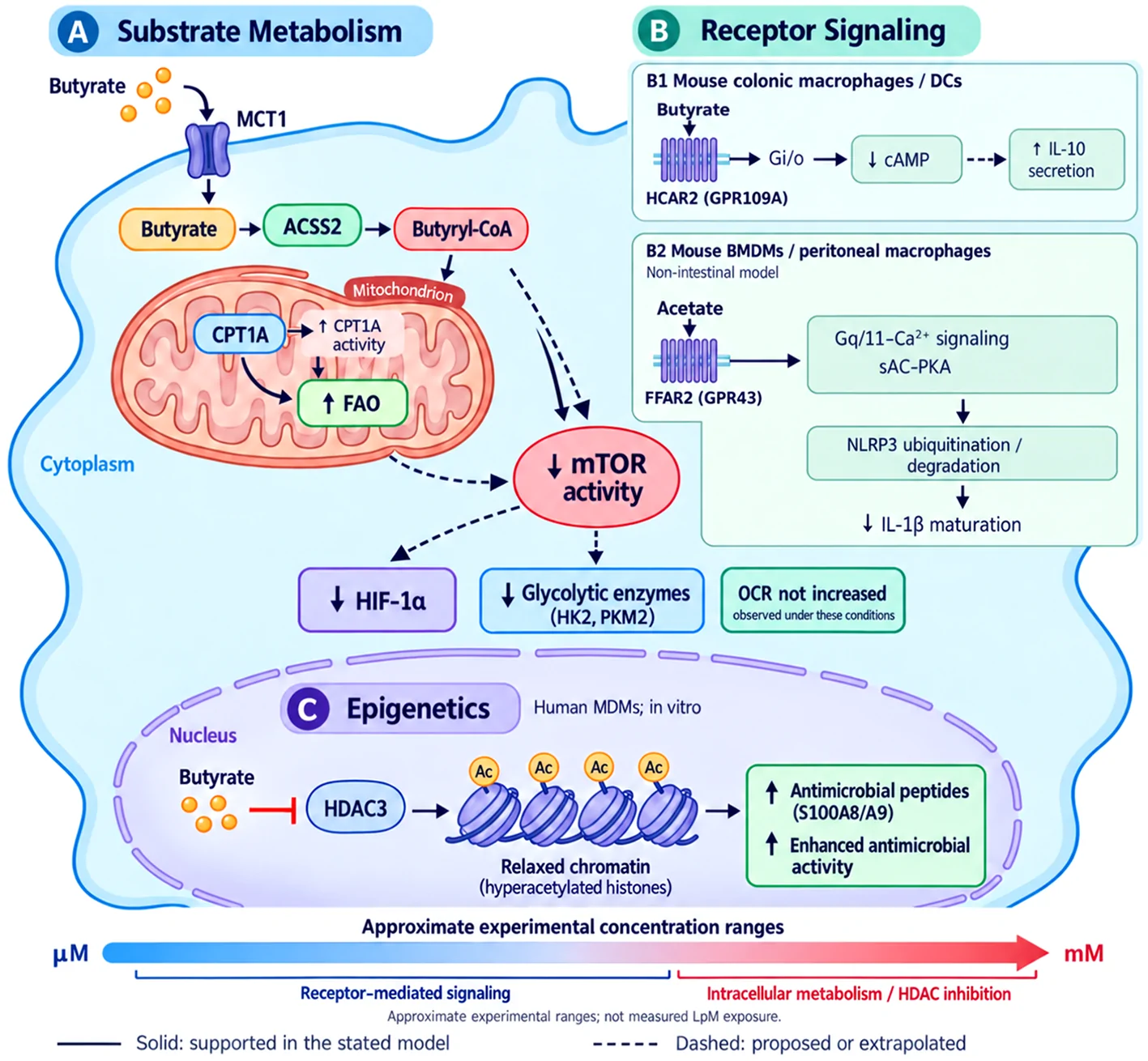
SCFA-responsive metabolic, receptor, and epigenetic pathways relevant to macrophage immunometabolism and their concentration-dependent context. **(A)** Summarizes intracellular butyrate metabolism, CPT1A/FAO-related pathways, and mTOR-associated metabolic regulation; **(B)** Shows HCAR2/GPR109A and FFAR2/GPR43 signaling; and **(C)** illustrates HDAC-dependent chromatin regulation. The BCoA–CPT1A interaction was demonstrated in mouse iTregs and has not been directly validated in intestinal LpMs. The concentration spectrum indicates approximate experimental domains rather than fixed biological thresholds: receptor-mediated signaling is often detected from micromolar to low-millimolar ranges, whereas intracellular metabolism and HDAC inhibition become increasingly relevant at millimolar concentrations. These ranges overlap and do not represent measured SCFA exposure at the LpM surface. Solid lines indicate relationships demonstrated in the stated experimental model, whereas dashed lines indicate proposed links between pathways.

**Table 2:** Representative mechanistic studies of SCFA regulation of macrophage immunometabolism, experimental exposure, and evidence in intestinal lamina propria macrophages.

Mechanism and study	Experimental model	SCFA/ligand concentration and treatment schedule	Readout	Main finding	Relation to intestinal LpMs
BCoA binding to CPT1A (72)	Mouse CD4+ iTreg cells	Butyrate cellular assays: 125 μM during iTreg polarization; CPT1 activity at 24 h and iTreg differentiation at 72 h. BCoA cellular assays: 125 μM for CPT1 activity at 24 h and iTreg differentiation at 72 h, including rescue experiments with cerulenin or an ACSS2 inhibitor. OCR assays: after 24-h iTreg polarization, 125 μM butyrate in XF Base Medium for 15–20 min before measurement. Cell-free assays: mitochondria/cell lysates from 24-h iTreg cultures incubated with BCoA (125 μM) for 5 min; recombinant CPT1A incubated for 5 min with malonyl-CoA (80 μM) and graded BCoA concentrations.	Cellular assays: CPT1 activity, OCR, and Foxp3+ iTreg differentiation. Cell-free assays: CPT1A activity; biolayer interferometry and Arg243 mutagenesis supported the BCoA–CPT1A interaction.	BCoA binds CPT1A and antagonizes malonyl-CoA-mediated inhibition, increasing CPT1A activity; the butyrate–BCoA–CPT1A axis supports FAO and iTreg differentiation.	Biochemical mechanism established in iTregs; it provides a candidate pathway for intestinal macrophage metabolism.
mTOR and glycolysis (65)	Human monocyte-derived macrophages; mouse intestinal macrophages *in vivo*	Human MDMs: butyrate, 1 mM throughout 5-day M-CSF differentiation. Mice: sodium butyrate, 150 mM in drinking water for 7 days before intestinal macrophage isolation.	Human MDMs: ECAR, metabolomics, energy charge, AMP/ATP ratio, AMPK/mTOR activity, LC3-associated host defense, and antimicrobial peptide expression. Mouse intestinal macrophages: ex vivo antimicrobial activity.	Butyrate lowers glycolytic activity and mTOR signaling and promotes an antimicrobial macrophage program.	Metabolic measurements were obtained mainly in human MDMs, with related antimicrobial effects observed in intestinal macrophages from butyrate-treated mice.
HDAC-dependent regulation (59)	Mouse BMDMs and sorted mouse colonic LP macrophages	Sorted colonic LP macrophages: n-butyrate, 500 μM with LPS (100 ng/mL); qPCR at 4 h and cytokine ELISA at 24 h. BMDMs: n-butyrate, 100 μM–2 mM with LPS; qPCR at 4 h and cytokine/NO assays at 24 h. Histone H3 acetylation: 1 μM–2 mM n-butyrate with LPS for 8 h. Mice: metronidazole/vancomycin for 10 days, followed by 300 mM n-butyrate with antibiotics in drinking water for 7 days.	Sorted colonic LP macrophages: inflammatory gene expression and cytokine secretion. BMDMs: inflammatory transcripts, cytokines, NO, and histone H3 acetylation. *In vivo*: qPCR in isolated colonic LP macrophages.	Butyrate suppresses selected inflammatory mediators in colonic LP macrophages and increases histone acetylation in BMDMs.	Functional effects were demonstrated in sorted mouse colonic LP macrophages; detailed chromatin and HDAC perturbation was characterized mainly in BMDMs.
FFAR2/GPR43 and NLRP3 (66)	Mouse BMDMs and peritoneal macrophages	Inflammasome assays: LPS (100 ng/mL) priming for 3 h; ATP (5 mM) or nigericin (20 μM) stimulation for 30 min. Dose-response assays used acetate, 20–60 mM. Timing experiments in BMDMs: acetate, 10–30 mM added 30 min before LPS, 30 min before nigericin, together with nigericin, or 30 min after nigericin. Mechanistic assays in LPS-primed BMDMs: 30-min acetate pretreatment; sAC/PKA experiments used 20 mM acetate. Ca2+ imaging in peritoneal macrophages: LPS priming for 3 h, followed by 20 mM acetate for 30 min before nigericin stimulation.	Dose-response assays: IL-1β and IL-18 secretion. Timing experiments: IL-1β, IL-18, TNF-α, and IL-6 secretion. BMDM mechanistic assays: GPR43/Gq perturbation, sAC/PKA dependence, and NLRP3 ubiquitination/degradation. Peritoneal macrophages: Ca2+ dynamics.	Acetate suppresses NLRP3 activation through FFAR2-dependent Ca2+ signaling and sAC-PKA-mediated NLRP3 ubiquitination/degradation.	Defines an acetate-responsive FFAR2-NLRP3 pathway in macrophages; intestinal relevance is supported conceptually but has not been tested directly in LpMs.
ACSS2, HIF-1α, and glycolysis (70)	Mouse BMDMs; mouse peritoneal macrophages for siRNA experiments	BMDM cytokine assays: acetate, 10 mM for 30 min before LPS (100 ng/mL), followed by 6 h of LPS stimulation. BMDM signaling/metabolic assays with 10 mM acetate and LPS: HIF-1α hydroxylation at 15 min, acetyl-CoA at 30 min, ECAR/OCR after 1 h, and glucose/lactate assays after 6 h of LPS stimulation. Peritoneal macrophages: *Ffar3* (GPR41)/*Ffar2* (GPR43) siRNA transfection for 36–48 h before acetate/LPS stimulation; *Acss2* siRNA transfection for 48 h before stimulation.	BMDMs: HIF-1α hydroxylation, acetyl-CoA, ECAR/OCR, glucose/lactate measurements, and cytokine responses with ACSS2 inhibition. Peritoneal macrophages: cytokine responses after *Acss2*, *Ffar3* (GPR41), or *Ffar2* (GPR43) silencing.	Acetate suppresses HIF-1α-dependent glycolysis through ACSS2 rather than FFAR2 or FFAR3.	Defines an intracellular acetate-metabolism pathway in BMDMs that may contribute to glycolytic regulation in intestinal macrophages.
Propionate and macrophage responses (67)	Mouse BMDMs	Microbial-response assays: propionate, 0.06–4 mM for 1 h before stimulation; inflammatory mRNA at 4 h, cytokine secretion at 8 h, and nitrite/nitrate at 24 h after stimulation. Epigenetic assays: propionate, 0–4 mM; *Hdac* transcripts after 4 or 18 h and histone H3/H4 acetylation after 18 h.	Microbial-response assays: qPCR, cytokine ELISA/Luminex, and Griess assay for NO products. Epigenetic assays: *Hdac* transcripts and acetylated histone H3/H4.	Propionate alters cytokine and nitric oxide responses and increases histone acetylation in a stimulus-dependent manner.	Supports an HDAC-related propionate effect in macrophages; intestinal macrophage data remain limited.
HCAR2/GPR109A in colonic APCs (60)	Mouse colonic LP macrophages and dendritic cells; splenic APC ligand-response assays	Splenic macrophages/DCs: butyrate or niacin, 0.5 mM for 2 days before *Il10*/*Aldh1a1* analysis or T-cell co-culture. Colonic LP macrophages/DCs from WT and *Gpr109a*-deficient mice: 4-day co-culture with naïve OT-II T cells; cytokines after overnight rest and 24-h T-cell restimulation. Mice: niacin, 25 mM with antibiotics in drinking water for 4 weeks.	Splenic APCs: *Il10*/*Aldh1a1* expression and Treg-inducing capacity. Colonic LP APCs: Foxp3+ Treg induction and T-cell IL-10/IL-17 secretion. Niacin-treated mice: Foxp3+ colonic CD4+ T cells.	GPR109A signaling supports anti-inflammatory and Treg-inducing functions in colonic antigen-presenting cells.	Provides direct functional evidence for GPR109A in mouse colonic LP macrophages and dendritic cells; defined ligand-dose assays were mainly performed in splenic APCs.
HCAR2/GPR109A and NLRP3 (73)	*Gpr109a*-deficient mice in caerulein pancreatitis; mouse peritoneal macrophages	Mice: sodium butyrate, 200 mg/kg intragastrically for 7 days before caerulein challenge; hourly caerulein injections for 10 h, with colonic tissue collected 1 h after the last injection. Peritoneal macrophages: sodium butyrate, 5 μM, 100 μM, or 2 mM for 24 h, followed by LPS (100 ng/mL) for 6 h.	Mice: colonic NLRP3-related proteins and GPR109A dependence. Peritoneal macrophages: HDAC1 expression, AP-1/STAT1 phosphorylation, and NLRP3-associated proteins.	Butyrate suppresses colonic NLRP3 signaling in a GPR109A-dependent manner; peritoneal macrophage experiments identify an HDAC1-AP-1/STAT1 pathway.	Links GPR109A to colonic NLRP3 regulation at tissue level, with macrophage-specific HDAC1-AP-1/STAT1 mechanisms characterized in peritoneal macrophages.

SCFA, short-chain fatty acid; LpM, lamina propria macrophage; BMDM, bone marrow-derived macrophage; MDM, monocyte-derived macrophage; BCoA, butyryl-CoA; CPT1A, carnitine palmitoyltransferase 1A; FAO, fatty acid oxidation; OCR, oxygen consumption rate; ECAR, extracellular acidification rate; HIF-1α, hypoxia-inducible factor 1α; HDAC, histone deacetylase; FFAR2/GPR43, free fatty acid receptor 2; HCAR2/GPR109A, hydroxycarboxylic acid receptor 2; APC, antigen-presenting cell; LP, lamina propria; iTreg, induced regulatory T cell; DC, dendritic cell; WT, wild type; LPS, lipopolysaccharide; NO, nitric oxide; sAC, soluble adenylyl cyclase; PKA, protein kinase A. Concentrations and treatment durations refer to the experimental conditions and do not represent measured SCFA exposure at the LpM surface. Post-stimulation times are measured from stimulus addition.

### 4.1 Intracellular SCFA metabolism and fatty acid oxidation

Hao et al. (72) defined a biochemical link between butyrate metabolism and fatty acid oxidation in mouse CD4+ induced regulatory T cells (iTregs). ACSS2 converted butyrate to butyryl-CoA (BCoA), and recombinant protein assays, biolayer interferometry, and Arg243 mutagenesis showed direct BCoA binding to CPT1A with reduced malonyl-CoA-mediated inhibition (72). OCR and long-chain acyl-carnitine measurements were consistent with increased fatty acid oxidation in this model (72).

Macrophages also rely on CPT1A-dependent fatty acid oxidation, and SCFAs can alter mitochondrial metabolism in macrophage systems (65, 70, 74). The BCoA-CPT1A interaction therefore provides a plausible route by which butyrate metabolism could influence macrophage bioenergetics, although this specific binding mechanism has so far been characterized in iTregs rather than intestinal macrophages (72).

### 4.2 mTOR, glycolysis, and mitochondrial function

During monocyte-to-macrophage differentiation, butyrate reduces glycolytic activity and mTOR signaling (65). Schulthess et al. (65) reported a lower extracellular acidification rate (ECAR), altered metabolite profiles, reduced cellular energy charge, an increased AMP/ATP ratio, greater AMPK phosphorylation, and lower mTOR kinase activity in butyrate-conditioned macrophages. These metabolic changes were linked to LC3-associated host defense and antimicrobial peptide production, connecting nutrient sensing with macrophage antimicrobial function (65). The same data also illustrate why antimicrobial activity, reduced inflammatory cytokine production, tissue tolerance, and M2-associated polarization should be viewed as related but distinct macrophage programs rather than a single functional state (15, 59, 65, 75).

Inflammatory macrophage glycolysis is embedded within broader remodeling of the tricarboxylic acid (TCA) cycle. A prominent feature of inflammatory activation is succinate accumulation, which stabilizes HIF-1α and promotes IL-1β production, thereby linking mitochondrial metabolism to inflammatory gene expression (76). HIF-1α also coordinates glycolysis-associated enzymes, including HK2, PKM2, and LDHA, reinforcing glucose utilization and lactate production in activated macrophage systems. This wider immunometabolic framework helps place the butyrate-induced changes in energy charge, AMPK, and mTOR reported by Schulthess et al. (65) into context. Butyrate can therefore intersect with HIF-associated metabolism through more than one route, including AMPK-mTOR regulation and HDAC-dependent transcriptional effects. These mechanisms are supported across macrophage models, but the individual steps linking butyrate, mTOR, HIF-1α, and glycolytic enzymes have not been demonstrated as one linear pathway in intestinal LpMs (22, 65, 76).

Acetate provides a distinct example of HIF-related metabolic regulation. In mouse BMDMs, 10 mM acetate given 30 min before LPS suppressed HIF-1α-dependent glycolysis through an ACSS2-dependent mechanism (70). Receptor knockdown did not support a requirement for GPR41 or GPR43, and ECAR measurements together with ACSS2 and HIF perturbation pointed to an intracellular metabolic effect (70). Thus, SCFAs can converge on glycolytic control through different entry points: butyrate is strongly linked to energy charge, AMPK-mTOR regulation, and HDAC-dependent programs, whereas acetate can alter HIF-1α-dependent glycolysis through intracellular acetate metabolism. The balance among these mechanisms depends on substrate, concentration, inflammatory stimulus, and macrophage state.

### 4.3 Receptor signaling through FFAR2/GPR43, FFAR3/GPR41, and HCAR2/GPR109A

SCFAs signal through free fatty acid receptor 2 (FFAR2/GPR43), free fatty acid receptor 3 (FFAR3/GPR41), and hydroxycarboxylic acid receptor 2 (HCAR2/GPR109A) (77–79). Receptor expression and G-protein coupling vary by species and cell type (80, 81). FFAR3 mainly signals through Gi/o, whereas FFAR2 can signal through both Gi/o and Gq/11 (81). HCAR2 is also Gi/o-coupled and reduces membrane adenylyl cyclase activity (82, 83). FFAR2 responds strongly to acetate and propionate, FFAR3 preferentially responds to propionate and butyrate, and HCAR2 is a low-affinity butyrate receptor generally activated at millimolar concentrations (78, 81).

Acetate and propionate engage both receptor-dependent and intracellular pathways in macrophages (66–71). In mouse BMDMs and peritoneal macrophages, acetate reduced NLRP3 activation through FFAR2/GPR43-dependent Gq/11-Ca2+ signaling, soluble adenylyl cyclase, PKA activity, and NLRP3 ubiquitination (66). In a separate BMDM study, acetate suppressed HIF-1α-dependent glycolysis through ACSS2 without a requirement for GPR41 or GPR43 (70). Propionate altered cytokine and nitric oxide responses and increased histone acetylation in mouse BMDMs, where *Ffar2* and *Ffar3* transcripts were not detected (67). Acetate or propionate also enhanced type I interferon responses in BMDMs and Peyer’s patch macrophages during enteric viral infection (68), while a propionate-producing engineered probiotic promoted anti-inflammatory macrophage responses in murine colitis (69). Conversely, propionate and butyrate activated NLRP3 in TLR-stimulated human macrophages in another setting (71), emphasizing the importance of dose, stimulus, and macrophage state.

HCAR2/GPR109A provides another route by which butyrate can influence mucosal immunity. Pan et al. (73) linked GPR109A to suppression of colonic NLRP3 signaling at the tissue level, while macrophage experiments in the same study implicated an HDAC1-AP-1/STAT1 pathway. Singh et al. (60) showed that *Gpr109a* signaling promotes anti-inflammatory and Treg-inducing functions in mouse colonic macrophages and dendritic cells. Together, these studies support HCAR2 as an immunoregulatory receptor in colonic antigen-presenting cells, with downstream pathways that vary across cell type and disease model.

FFAR2/GPR43 signaling is similarly context dependent (66, 71, 84). The acetate-NLRP3 study directly implicated GPR43-dependent Ca2+ signaling and soluble adenylyl cyclase/PKA activity (66), whereas other macrophage and non-macrophage systems show different downstream responses (66, 71). FFAR3/GPR41 is less well characterized in macrophages, and much of its current immunological context comes from other immune and non-immune cell types (85).

### 4.4 HDAC inhibition and histone modification

HDAC inhibition is one of the best-supported SCFA mechanisms in intestinal macrophages (59, 65). Chang et al. (59) showed that 500 μM n-butyrate reduced selected LPS-induced inflammatory mediators in sorted mouse colonic LP macrophages *in vitro*. In antibiotic-treated mice, 300 mM n-butyrate in drinking water for 7 days also reduced *Il6*, *Nos2*, *Il12a*, and *Il12b* expression in isolated colonic LP macrophages (59). In BMDMs from the same study, butyrate increased histone H3 acetylation and reproduced the effects of HDAC inhibition without requiring *Gpr109a* or *Gpr43* (59). These findings support an intracellular HDAC-dependent mechanism (59).

HDAC-dependent effects also vary with macrophage function and inflammatory context (59, 65, 86). Chang et al. (59) showed selective suppression of inflammatory mediators, Schulthess et al. (65) linked HDAC3 inhibition to LC3-associated host defense and antimicrobial peptide production, and Chen et al. (86) described HDAC3-dependent ROS-mediated bactericidal activity in an infection model. These observations illustrate that SCFA-induced epigenetic regulation can support distinct macrophage functions rather than a single polarization state.

Butyrate also promotes Foxp3-associated Treg differentiation through histone acetylation in CD4+ T cells (61), providing an additional route by which microbial metabolites can shape mucosal immune tolerance. In mouse colonic LP macrophages, HDAC-dependent regulation of inflammatory gene expression has been demonstrated directly (59). Recent human mucosal and monocyte/macrophage studies further implicate HDAC3, although the dominant HDAC isoforms, target genes, and chromatin-accessibility changes in human LpMs remain poorly defined (87).

### 4.5 Integration of metabolic, receptor, and epigenetic pathways

SCFA metabolism, receptor signaling, and HDAC-dependent regulation are interconnected rather than isolated pathways. Changes in cellular energy status can influence AMPK-mTOR signaling, receptor activation can reshape inflammatory transcription, and intracellular SCFA metabolism supplies acetyl-CoA and other acyl-CoA pools that can influence chromatin regulation (59, 65, 66, 70, 72, 88, 89). Butyrate provides the clearest example of this integration because it can alter macrophage metabolism, engage HCAR2, and inhibit HDAC activity, while acetate and propionate add FFAR2/FFAR3 signaling, ACSS2-dependent metabolism, inflammasome regulation, and histone acetylation (59, 60, 66, 67, 70). **Table 2** summarizes representative mechanistic studies, their experimental conditions, and their relevance to intestinal LpMs.

Concentration is an important part of this integration. At lower experimental concentrations, receptor-mediated responses through FFAR2 and FFAR3 can contribute prominently, depending on ligand, receptor expression, and assay conditions; PPARγ-associated programs may further modify macrophage responses in selected settings (37, 38, 75, 77, 81). HCAR2 is a lower-affinity butyrate receptor and is generally engaged at millimolar concentrations (78, 81). As extracellular SCFA concentrations rise into the millimolar range, cellular uptake, intracellular metabolism, and HDAC inhibition become increasingly important, so receptor-mediated and intracellular mechanisms may operate in parallel rather than in separate concentration compartments. The micromolar-to-millimolar spectrum is therefore best viewed as a conceptual summary of experimental systems rather than a measured LpM exposure threshold (28, 29).

The concentration spectrum also helps explain apparently bidirectional SCFA effects. Macrophage studies have reported anti-inflammatory and proinflammatory responses to butyrate at different concentrations and activation states (71, 90), while blood-pressure studies show that SCFA receptor systems can generate opposing physiological effects according to receptor affinity and ligand exposure (91, 92). This concentration dependence provides a pharmacological rationale for controlled intestinal delivery, but it also argues against extrapolating culture concentrations directly to the mucosa. Formulation, epithelial absorption, first-pass metabolism, and the concentration that actually reaches the lamina propria are therefore central considerations for precision delivery of SCFA-based interventions (28, 29, 93).

## 5 SCFAs, intestinal macrophages, and metabolic syndrome phenotypes

### 5.1 SCFA-macrophage pathways across metabolic phenotypes

#### 5.1.1 Central obesity and adipose tissue inflammation

Obesity is accompanied by changes in the number, phenotype, and metabolic programs of colonic macrophages (21–23). These mucosal changes occur alongside barrier dysfunction, metabolic endotoxemia, and adipose tissue inflammation, creating several routes through which intestinal immune remodeling may influence systemic metabolism (21, 22, 44, 46).

Rohm et al. (22) identified mTOR as a regulatory node associated with inflammatory and mitochondrial programs in colonic macrophages. TREM2-dependent lipid handling has been characterized in lipid-associated macrophage populations and provides a broader framework for macrophage adaptation to lipid-rich environments (37, 38). FFAR2/GPR43 signaling also shapes macrophage responses in adipose tissue, where receptor activation can modify inflammatory output in M2-polarized cells (94). These findings emphasize that SCFA-responsive pathways are shared across metabolic tissues but are shaped by the local macrophage niche and ligand availability.

#### 5.1.2 Insulin resistance and type 2 diabetes

In HFD-fed mice, colonic macrophage activation is accompanied by interferon-response and mitochondrial metabolic signatures (22). Local colonic mTOR inhibition reduces inflammatory macrophage abundance and improves metabolic measures, while rectal clodronate depletion of colonic macrophages improves glucose tolerance and insulin sensitivity (22). HIF-1α-dependent glycolytic programs are well established in activated macrophages (76), and the combined data place mTOR-dependent metabolic remodeling among the pathways linking intestinal inflammation with insulin resistance. The detailed sequence connecting mTOR, HIF-1α, and glycolytic flux in LpMs remains incompletely defined.

Inflammatory cytokines can impair insulin signaling through IRS-1/PI3K/Akt pathways (44). SCFAs also influence glucose homeostasis through several cell types, including macrophages and enteroendocrine cells (58, 62, 63). Butyrate reduces glycolytic activity and mTOR signaling in macrophages (65), whereas FFAR2 and FFAR3 signaling in intestinal L cells stimulates GLP-1 secretion (95). In a 4-week exploratory study, oral sodium butyrate (4 g/day) improved peripheral and hepatic insulin sensitivity in lean men but not in men with MetS (96), indicating that baseline metabolic state can modify the response to SCFA supplementation.

#### 5.1.3 Dyslipidemia and MetS-associated MASLD

MASLD is strongly associated with obesity, insulin resistance, and dyslipidemia and is closely linked to the gut-liver axis (2, 4). During disease progression, resident Kupffer cell programs change and monocyte-derived macrophages accumulate in the liver (39, 40). Portal microbial products, including LPS, can reach the liver and activate inflammatory pathways, while changes in microbial SCFA production can influence both intestinal and hepatic metabolism (10, 40).

SCFA-related mechanisms in MASLD have been studied mainly in hepatic macrophages, whole-liver models, and microbiota-directed experiments (39, 40, 97, 98). These studies implicate PPARγ-associated macrophage states, HDAC-dependent inflammatory regulation, and butyrate-responsive pathways in the control of steatosis and inflammation (39, 97, 98). Intestinal macrophages may contribute indirectly through barrier maintenance and regulation of portal inflammatory signals, but their specific contribution to the hepatic response remains unresolved.

Clinical studies further associate MASLD with gut dysbiosis and altered SCFA profiles (40). These observations support a network in which epithelial barrier function, enteroendocrine signaling, portal SCFA delivery, intestinal macrophages, and Kupffer cells interact along the gut-liver axis. Defining how these compartments communicate will be important for separating local intestinal effects from direct hepatic actions of SCFAs.

#### 5.1.4 Hypertension and vascular effects

SCFAs influence blood pressure through vascular, renal, endocrine, and intestinal mechanisms (91, 92, 99). *Gpr41*/*Gpr43* double-knockout mice develop greater cardiac and renal fibrosis with increased renal macrophage infiltration, linking host SCFA sensing to target-organ inflammation (99). At the intestinal level, preservation of barrier integrity may also reduce systemic LPS exposure and vascular inflammatory tone (44, 46).

At peripheral sites, SCFAs regulate vascular and renal physiology through receptors including FFAR3/GPR41 and Olfr78 (91, 92). Propionate activates these receptors at different concentrations and can produce opposing effects on vascular tone and renin release, illustrating the concentration-dependent nature of SCFA signaling in blood-pressure regulation (91, 92).

Human studies likewise show heterogeneous blood-pressure responses to SCFA-related interventions. In a phase II double-blind crossover trial, 40 g/day HAMSAB for 3 weeks increased circulating acetate and butyrate and reduced 24-hour systolic blood pressure in untreated hypertension (100). A proof-of-concept crossover study found lower daytime systolic blood pressure after an 80 mmol/L butyrate enema (101). By contrast, a 4-week randomized placebo-controlled trial in 23 patients with hypertension found that oral sodium butyrate (3.9 g/day) increased daytime systolic and diastolic blood pressure (102). These contrasting results suggest that formulation, delivery route, dose, and host metabolic context all influence the vascular response to SCFAs.

#### 5.1.5 Integration across MetS-associated phenotypes

Across metabolic phenotypes, SCFAs intersect with macrophage biology at different anatomical levels. Obesity and insulin resistance provide the clearest evidence for colonic macrophage recruitment and metabolic remodeling (21, 22), whereas MASLD emphasizes gut-liver communication and hepatic macrophage responses (39, 40). Hypertension highlights vascular and renal SCFA sensing together with barrier-dependent control of systemic inflammatory exposure (91, 92, 99–102). These tissue-specific patterns argue against a single SCFA-macrophage mechanism for all components of MetS.

From a therapeutic perspective, the same diversity creates both opportunities and challenges. Dietary fiber, microbiota manipulation, exogenous SCFAs, and bariatric surgery can alter metabolic outcomes through overlapping effects on microbial ecology, epithelial physiology, endocrine signaling, and immune function (93, 103–105). A clearer understanding of tissue exposure and macrophage-specific responses will help identify which patients and metabolic phenotypes are most likely to benefit from interventions targeting the microbiota-SCFA axis. **Figure 4** summarizes the dominant pathways, evidence strength, and key evidence gaps regarding SCFA regulation of intestinal macrophage immunometabolism across obesity, insulin resistance, MASLD, and hypertension.

**Figure 4:**
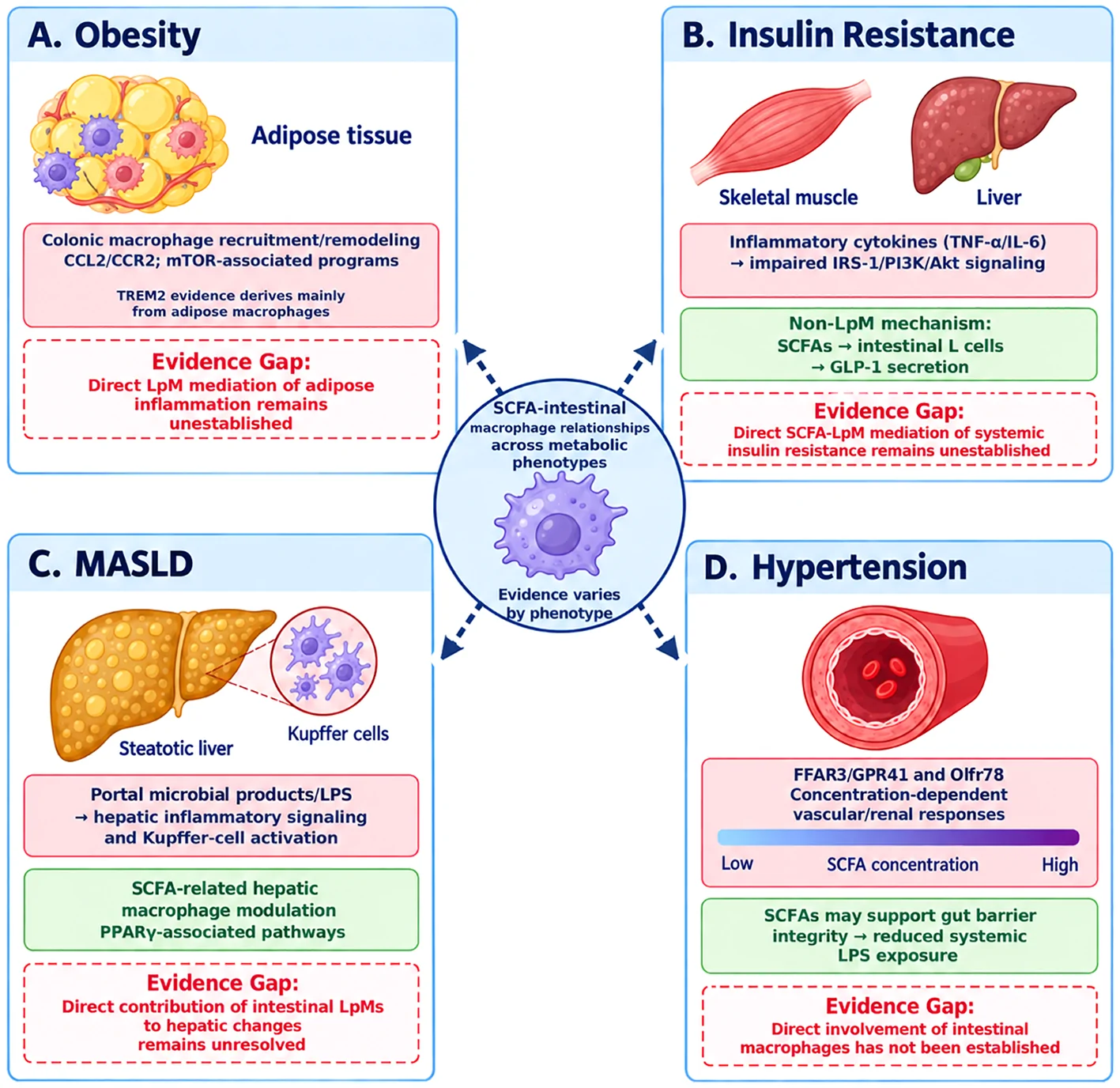
Tissue-specific relationships among SCFAs, intestinal macrophages, and metabolic phenotypes. **(A)** Obesity and **(B)** insulin resistance emphasize colonic macrophage remodeling; **(C)** MASLD highlights gut-liver and hepatic macrophage pathways; and **(D)** hypertension involves vascular and renal SCFA sensing in addition to intestinal barrier effects. Dashed arrows indicate cross-tissue or proposed relationships. Dashed boxes mark evidence gaps where direct LpM involvement has not been established.

### 5.2 Clinical interventions targeting the microbiota-SCFA axis

Human interventions that modify diet, microbiota composition, SCFA production, or SCFA delivery can influence cardiometabolic outcomes (93, 100, 103–105). Clinical studies are therefore useful for assessing the translational potential of the microbiota-SCFA axis, although their effects usually reflect several intestinal and systemic pathways operating together. Representative human intervention studies, including dietary fiber, FMT, probiotics, exogenous SCFA delivery, and bariatric surgery, are summarized in **Supplementary Table S1**.

#### 5.2.1 Dietary fiber and fermentable carbohydrates

Dietary-fiber trials generally support cardiometabolic benefits, although the magnitude and metabolic profile of the response vary among individuals (103, 106–109). In a double-blind crossover study, 16 adults at cardiometabolic risk were randomized and 15 completed the study; chitin-glucan (4.5 g/day for 3 weeks) altered the gut microbiota and reduced postprandial triglyceridemia, while lower postprandial glycemia was observed mainly in participants with high baseline H2 production (103). Fecal SCFA concentrations did not differ from control (103). Other trials show that structured provision of fiber-enriched foods increases habitual fiber intake (106), while oat interventions link cholesterol lowering to microbially generated phenolic metabolites (107). These findings emphasize that fiber can modify cardiometabolic physiology through several microbial products rather than through SCFAs alone.

Meta-analyses extend these observations across broader populations. Soluble and viscous fibers improve serum lipid profiles and glycemic control, and psyllium supplementation can influence body weight and other anthropometric measures (108, 109). The response depends partly on baseline microbiota composition and fermentation capacity, which may explain why similar fiber doses produce different SCFA profiles and metabolic outcomes among individuals. Combining dietary records with fecal, circulating, and, where possible, tissue metabolite measurements should therefore improve interpretation of fiber-based interventions.

#### 5.2.2 Exogenous SCFAs and colon-targeted pharmacokinetics

Direct SCFA delivery bypasses microbial production but is strongly influenced by formulation and the site of administration (29, 93). In a randomized crossover study of 28 healthy adults, delivery of the same SCFA dose to the small intestine or colon produced different circulating SCFA concentrations and enteroendocrine responses (93). Stable-isotope studies likewise show substantial first-pass utilization of colonic SCFAs before they enter the systemic circulation (29). These pharmacokinetic data provide a rationale for pH-dependent or other colon-targeted formulations and for rectal delivery when high local exposure is required, although convenience, tolerability, epithelial uptake, and mucosal penetration remain practical constraints. The relevant pharmacological variable is therefore not the administered dose alone, but the amount and duration of SCFA exposure within the colonic mucosa.

#### 5.2.3 Probiotics and fecal microbiota transplantation

Probiotic and synbiotic interventions can modify microbiota composition, SCFA production, and metabolic outcomes (104, 110). In a randomized double-blind placebo-controlled study of 60 participants at high risk of MetS, 6 months of Lacticaseibacillus casei LC2W improved several metabolic and inflammatory measures, with increased fecal SCFAs observed at 3 months; 55 participants completed the study (104). A separate randomized trial in adults with MetS used 300 g/day of synbiotic yogurt for 12 weeks and reported improvements in fasting glucose, insulin resistance, waist-to-hip ratio, and systolic blood pressure; the formulation contained Lactobacillus plantarum, Lactobacillus pentosus, and a yeast strain together with prebiotic ingredients (110). These studies illustrate how probiotic effects can extend beyond a single metabolite and depend on the strain combination, diet, and host microbial ecosystem.

FMT provides a broader approach to reshaping the gut microbial ecosystem and can influence insulin sensitivity, plasma metabolites, and host immune epigenetic profiles (55, 56, 64, 111–113). Lean-donor FMT improved insulin sensitivity in the Vrieze study (55), while later work showed that recipient microbiome features predict metabolic response (111) and that FMT can alter DNA methylation patterns in participants with MetS (112). Donor metabolic status is also important: transfer from donors with different metabolic phenotypes produced different effects on recipient insulin sensitivity, energy expenditure, and intestinal transit (113). Together, these findings make donor-recipient compatibility and functional engraftment central considerations for metabolic FMT.

#### 5.2.4 Bariatric surgery and metabolic modulators

Bariatric surgery produces broad metabolic, hormonal, immune, and microbiome changes in addition to weight loss (105, 114–119). In a prospective study of 24 patients with severe obesity, sleeve gastrectomy and gastric bypass produced similar clinical improvement at 6 months but distinct gut microbiome profiles and peripheral immune gene expression (105). Across studies, surgery also alters gut hormone signaling, bile-acid metabolism, nutrient flow, branched-chain amino-acid metabolism, and microbial ecology, creating several routes through which SCFA production and immune function may change (114–117). These systemic effects make bariatric surgery an informative model of microbiota-host metabolic remodeling, but they also make it difficult to attribute clinical benefit to any single SCFA-responsive cell population.

## 6 Discussion

### 6.1 Animal models and translational relevance

The mechanistic evidence reviewed above derives from several distinct experimental systems. Diet-induced obesity models allow *in vivo* assessment of colonic macrophage recruitment, inflammatory remodeling, and associated metabolic phenotypes (21–23), whereas receptor-deficient mice have been used to define the contribution of specific SCFA-sensing pathways (59, 65, 73). Germ-free and gnotobiotic mice provide controlled systems for examining host–microbiota interactions and the effects of defined microorganisms or microbial communities on host immune and metabolic phenotypes (120). These models are particularly useful for testing causal relationships between microbial exposure and host responses, although experimental outcomes are influenced by microbial composition, host genetic background, diet, housing conditions, and study design (120, 121). Different preclinical microbiome models therefore capture distinct aspects of host–microbiota biology rather than the full complexity of the human intestinal environment (121). Similar limitations apply to models of metabolic disease, in which differences in physiology, disease induction, and experimental context can restrict direct extrapolation from mice to humans (122). Murine BMDMs and peritoneal macrophages provide complementary systems for detailed analysis of SCFA-responsive metabolic and signaling pathways, but they do not reproduce the cellular composition or local metabolic conditions of the intestinal lamina propria. Human studies have mainly characterized intestinal macrophage phenotypes, transcriptional states, and tissue organization (16–20), while iPSC-derived macrophages and pluripotent-stem-cell-derived intestinal organoid models incorporating tissue-resident macrophages provide human-relevant experimental systems for further mechanistic studies (25, 26).

### 6.2 SCFA exposure and pharmacokinetic considerations

Beyond differences among experimental models, SCFA pharmacokinetics introduce an additional source of translational variability. Fecal, luminal, mucosal/interstitial, portal, and peripheral measurements represent different compartments, and delivery route determines how much SCFA is absorbed, metabolized by the epithelium, or reaches the mucosa and circulation (27–29, 32, 33, 51, 93). Macrophage cultures also show different inflammatory responses across SCFA concentrations and activation states (71, 90). Together, these observations suggest that future therapeutic development will require attention to formulation, anatomical delivery, local exposure, and patient-specific microbial fermentation rather than reliance on a single systemic or fecal concentration.

### 6.3 Future research

Future work should combine human-relevant macrophage models with spatially resolved measurements of the intestinal environment. iPSC-derived intestinal macrophages and enteroid–immune cell co-cultures can be integrated with controlled gradient platforms to model SCFA exposure, oxygen tension, and epithelial transport (25, 26, 123). Spatial transcriptomics, mass spectrometry imaging, and interstitial fluid sampling can relate macrophage states to local metabolite abundance (28, 124, 125). In clinical intervention studies, pairing colon biopsies with macrophage-specific readouts such as mTOR activity, HDAC-dependent chromatin changes, receptor-proximal signaling, cytokine responses, and, where feasible, ECAR, OCR, or isotope-based flux measurements would provide a more direct view of how diet, microbial metabolites, and intestinal immunity interact in humans.

## 7 Conclusion

This narrative review synthesizes current evidence on how SCFAs regulate LpM immunometabolism through substrate metabolism, GPCR signaling, and HDAC inhibition, and evaluates the contribution of this axis across MetS-associated phenotypes. LpMs, with their tolerance phenotype and anatomical localization, are well positioned to link microbiota-derived metabolites with intestinal and systemic immune regulation. The strength and location of evidence nevertheless differ across mechanisms: AMPK–mTOR–glycolysis remodeling is well characterized in macrophage systems and supported by intestinal functional studies, although the complete mTOR–HIF-1α–glycolysis sequence has not been established in LpMs; HCAR2 has direct functional support in mouse colonic macrophages and dendritic cells; HDAC-dependent suppression of inflammatory mediators has been demonstrated in sorted mouse colonic LP macrophages, while detailed chromatin mechanisms are defined mainly in BMDMs; and the BCoA–CPT1A mechanism derives primarily from iTreg studies, with much of the acetate and propionate work performed in BMDMs or other extraintestinal macrophages. Across MetS, intestinal macrophage remodeling is most clearly linked to obesity and insulin resistance, whereas MASLD and hypertension involve broader gut–liver, vascular, and renal networks. Human interventions produce heterogeneous metabolic effects, underscoring the importance of local exposure, formulation, and host metabolic state. Although a theoretical framework for the SCFA–LpM axis has been established, the field remains far from clinical application. Future studies should integrate human-derived cell models, spatially resolved SCFA measurements, and LpM-specific pharmacodynamic endpoints in intervention trials, thereby providing an actionable translational path for precision therapies targeting intestinal macrophages.
