# Supplementary material for: Short-chain fatty acids and intestinal macrophage immunometabolism in metabolic syndrome: mechanisms and implications for chronic low-grade inflammation
Source: Front Immunol. 2026 Oct 22;17:2000672. doi: 10.3389/fimmu.2026.2000672 (PMC13735788; doi:10.3389/fimmu.2026.2000672)
Supplement: Supplementary file 1 [file DataSheet1.pdf]

Supplementary Table S1. Representative human intervention studies involving SCFA-related strategies in metabolic syndrome and related cardiometabolic conditions.

Study and population	Intervention	Comparator	Follow-up	Main outcome	SCFA measurements and macrophage endpoints
Ranaivo et al. (103), 16 adults at cardiometabolic risk randomized; 15 completed and were analyzed	Chitin-glucan 4.5 g/day	Maltodextrin in a crossover design	3 weeks per arm	Postprandial triglyceridemia decreased; postprandial glycemia decreased only in the high-H_2 subgroup, and the microbiota changed.	Fecal SCFAs were measured; no significant between-treatment difference was found. Mucosal/interstitial exposure and LpM phenotype/function were not assessed.
Vrieze et al. (55), 18 men with MetS	Lean-donor microbiota infusion	Autologous microbiota	6 weeks	Peripheral insulin sensitivity increased and butyrate-producing taxa increased.	Fecal SCFAs were measured at baseline and 6 weeks. Mucosal/interstitial SCFA exposure and LpM phenotype/function were not assessed.
Piwchan et al. (56), 18 participants with MetS	FMT enema, n=8	Sham enema, n=10	6 and 12 weeks	HOMA-IR, FBG, and hs-CRP improved at 6 weeks. Effects were not consistent at 12 weeks.	Target-tissue SCFA exposure was not reported. LpM phenotype/function was not assessed.
Wang et al. (104), 60 participants at high risk of MetS, 55 completed	Lacticaseibacillus casei LC2W, 6×10^11 CFU/day	Maltodextrin placebo	6 months	Glucose, lipid, BMI, and inflammatory outcomes improved.	Fecal SCFAs were measured at baseline and 3 months; acetate, butyrate, and total SCFAs increased at 3 months. Mucosal/interstitial exposure and LpM phenotype/function were not assessed.
Lazaro et al. (105), 24 patients with severe obesity	Sleeve gastrectomy, n=14	Gastric bypass, n=10	6 months	Clinical improvement was similar after sleeve gastrectomy and gastric bypass at 6 months, but microbiome and peripheral immune gene expression profiles differed between procedures.	No direct SCFA measurement was reported. LpM phenotype/function was not assessed.
Jama et al. (100), 20 untreated participants with hypertension	HAMSAB 40 g/day	Placebo in a crossover design	3 weeks per arm with a 3-week washout	24-hour systolic blood pressure decreased.	Plasma acetate and butyrate increased. Local mucosal/interstitial exposure and LpM phenotype/function were not assessed.
Hogue et al. (101), 10 Black adults with stage 1 hypertension underwent crossover enema treatment; 10 normotensive participants served as a reference group	Butyrate enema, 80 mmol/L, 60 mL	Butyrate enema, 5 mmol/L, 60 mL in a crossover design	24-hour blood pressure monitoring. Doses were given 7 days apart.	Daytime systolic blood pressure was lower after 80 mmol/L.	Enema butyrate concentration and volume were specified. Blood butyrate did not increase significantly at 30 min after enema administration. Pericellular LpM exposure and LpM phenotype/function were not assessed.
Bouter et al. (96), 9 lean men and 10 men with MetS	Oral sodium butyrate 4 g/day	No placebo; within-group pre/post comparison	4 weeks	Peripheral and hepatic insulin sensitivity improved in lean participants but not in participants with MetS.	Plasma and fecal SCFAs were measured; butyrate did not increase in plasma or feces. Local mucosal/interstitial exposure and LpM phenotype/function were not assessed.
Verhaar et al. (102), 23 patients with hypertension randomized; 2 withdrew before the baseline visit	Oral sodium butyrate 3.9 g/day	Sodium-matched placebo (sodium chloride 2.0 g/day); double-blind randomized design	4 weeks	Daytime systolic and diastolic blood pressure, assessed by 24-hour ambulatory monitoring, increased versus placebo.	Plasma butyrate increased, whereas fecal butyrate did not. Local mucosal/interstitial exposure and LpM phenotype/function were not assessed.

Notes: SCFA, short-chain fatty acid; MetS, metabolic syndrome; FBG, fasting blood glucose; FMT, fecal microbiota transplantation; HAMSAB, acetylated and butyrylated high-amylose maize starch; LpM, lamina propria macrophage; HOMA-IR, homeostatic model assessment of insulin resistance; hs-CRP, high-sensitivity C-reactive protein; BMI, body mass index; CFU, colony-forming units; H_2, hydrogen. References correspond to the numbering used in the main manuscript.
